# A missense variant in EZH2 is associated with colorectal cancer risk in a Chinese population

**DOI:** 10.18632/oncotarget.21888

**Published:** 2017-10-16

**Authors:** Huihui Li, Chunxiao Chang, Yuhong Shang, Ling Qiang, Baoxuan Zhang, Bing Bu, Guohua Ren, Lihua Song, Mao Shang, Jinming Yu

**Affiliations:** ^1^ Shandong University, Jinan, Shandong Province, China; ^2^ Department of Medical Oncology, Shandong Cancer Hospital Affiliated to Shandong University, Shandong Academy of Medical Sciences, Jinan, Shandong Province, China; ^3^ Clinical Laboratory, Shandong Cancer Hospital Affiliated to Shandong University, Shandong Academy of Medical Sciences, Jinan, Shandong Province, China; ^4^ Department of Radiation Oncology, Shandong Cancer Hospital Affiliated to Shandong University, Shandong Academy of Medical Sciences, Jinan, Shandong Province, China

**Keywords:** colorectal cancer, susceptibility, enhancer of zeste 2 polycomb repressive complex 2 subunit, exon region, missense variant

## Abstract

Colorectal cancer (CRC) ranks the fifth leading cause of cancer death in China. EZH2 is a member of Polycomb-group (PcG) family and associated with transcriptional repression and cancer development. In this study, we report the association between a missense variant in EZH2 and risk of CRC. Through a systematic selection of variants in EZH2, we identified rs2302427 in the exon region of EZH2 and genotyped this variant in 852 CRC patients and 1,303 healthy controls using Taqman genotyping assay. The association between this variant and CRC risk was calculated using logistic regression with adjustment of sex, age, smoking status and drinking status. The result showed that rs2302427 was significantly associated with CRC susceptibility under an additive model (*P*=0.0068). Compared with CC genotype carriers, CG genotype and GG genotype carriers were associated with risk of CRC with odds ratio being 0.78 (95% CI: 0.63-0.96, *P*=0.0198) and 0.54 (95% CI: 0.24-1.18, *P*=0.1224), respectively. When stratified by sex, age, smoking status or drinking status, significant associations were observed only in younger individuals (OR=0.67, 95% CI: 0.50-0.89, *P*=0.0067) or smokers (OR=0.65, 95% CI: 0.48-0.88, *P*=0.0051). This study provides new insights into the personalized prevention of colorectal cancer.

## INTRODUCTION

Colorectal cancer (CRC) is one of the most common malignancies worldwide and ranks the third leading cause of cancer in both men and women [1-3]. Although environmental factors such as over-nutrition, obesity and smoking have been associated with the development of sporadic CRC [4, 5], only a small portion of exposed individuals develop CRC, indicating that an individual’s genetic makeup also play an important role in the etiology of CRC [6, 7]. Previous studies have identified many genetic polymorphisms that were correlated with colorectal cancer risk in the Chinese population using both genome-wide association [8, 9] and candidate strategy [10, 11].

Enhancer of zeste 2 polycomb repressive complex 2 subunit (EZH2), a histone methyl transferase subunit of polycomb repressor complex 2 (PRC2), a complex that methylates lysine 27 of histone H3 (H3K27) to promote transcriptional silencing [12, 13]. EZH2 is recurrently mutated in many types of cancer and play an important role in cancer proliferation and progression [14-16]. Polymorphisms in EZH2 have been identified to be associated with multiple cancers, such as bladder cancer, Oral squamous cell carcinoma and colorectal cancer [17-20]. However, most of these studies only focus on variants in the promoter region of EZH2, the association between missense variants in EZH2 and risk of colorectal cancer were not interrogated.

Here we report a case control study for a missense variant in EZH2 and colorectal cancer susceptibility. This study consists of 852 CRC patients and 1,303 controls and the result showed that rs2302427 was significantly associated with colorectal cancer risk.

## RESULTS

### Select characteristic of study subjects

The distributions of select characteristics including sex, age, smoking status and drinking status of the CRC patients and controls participated in this study were shown in Table 1. 852 CRC patients and 1,303 healthy controls were used in the analysis. There were 67.6% and 65.2% males in cases and controls, respectively. The average age of cases and controls were 60.7 and 63.5, respectively. There are 40.8% smokers and 30.2% drinkers among cases while there are 36.5% smokers and 29.2% drinkers among controls.

**Table 1:** Select characteristics of subjects participated in this study

	Cases (n = 852)	Controls (n = 1,303)
Age (years), mean ± S.D.	60.7 ± 13.1	63.5 ± 9.6
Gender, n (%)		
Male	576 (67.6)	850 (65.2)
Female	276 (32.4)	453 (34.8)
Smoking status		
Nonsmoker	504 (59.2)	827 (63.5)
Smoker	348 (40.8)	476 (36.5)
Drinking status		
Nondrinker	595 (69.8)	923 (70.8)
Drinker	257 (30.2)	380 (29.2)

### Association between rs2302427 and risk of CRC

We found only one variant located in the exon region of EZH2 with minor allele frequency > 1% in Chinese population. There are five variants that are in high LD (*r*^2^ > 0.6) with rs2302427. All these SNPs are in the intron region of EZH2 with no functional clue. The genotype frequencies of this variant and its association with risk of CRC were shown in Table 2. The rs2302427 was significantly associated with CRC susceptibility with odds ratio being 0.77 in an additive model (95% CI: 0.64-0.93, *P*=0.0068). Compared with CC genotype carriers, CG genotype and GG genotype carriers were associated with risk of CRC with odds ratio being 0.78 (95% CI: 0.63-0.96, *P*=0.0198) and 0.54 (95% CI: 0.24-1.18, *P*=0.1224), respectively.

**Table 2:** Association between rs2302427 and risk of CRC in a Chinese population

SNP	Chr	Position	Location	Genotype	Cases No. (%)	Controls No. (%)	OR (95% CI)^a^	*P*^a^
rs2302427	7	148525904	Asp>His	CC	670 (78.6)	961 (73.8)	1.00 (Reference)	
				CG	173 (20.3)	319 (24.5)	0.78 (0.63-0.96)	0.0198
				GG	9 (1.1)	23 (1.8)	0.54 (0.24-1.18)	0.1224
				CG+GG	182 (21.4)	342 (26.2)	0.76 (0.62-0.94)	0.0096
				Additive model			0.77 (0.64-0.93)	0.0068

^a^Calculated by logistic regression with adjustment for sex, age, smoking status and drinking status.

### Stratified analysis of rs2302427 and risk of CRC

We performed stratified analyses by sex, age, smoking status and drinking status to evaluate the effects of variant genotypes on the risk of CRC (Table 3). Among the younger individuals (≤ 60 years), rs2302427 was significantly associated with CRC susceptibility with an odds ratio of 0.67 (95% CI: 0.50-0.89, *P*= 0.0067). However, the association were not significant for older individuals (> 60 years) (OR= 0.85, 95% CI: 0.66-1.10, *P*=0.2175). When stratified by smoking status, rs2302427 was significantly associated with CRC risk in smokers, but not in nonsmokers, with the OR being 0.65 (95% CI: 0.48-0.88, *P*= 0.0051) and 0.87 (95% CI: 0.68-1.11, *P*=0.2508), respectively.

**Table 3:** Result of stratification analysis for rs2302427

	OR (95% CI)	*P*
Male	0.83 (0.66-1.04)	0.1130
Female	0.63 (0.44-0.90)	0.0102
> 60 years	0.85 (0.66-1.10)	0.2175
≤ 60 years	0.67 (0.50-0.89)	0.0067
Smoker	0.65 (0.48-0.88)	0.0051
Nonsmoker	0.87 (0.68-1.11)	0.2508
Drinker	0.78 (0.54-1.12)	0.1720
Nondrinker	0.76 (0.60-0.95)	0.0149

### Functional prediction of rs2302427

The rs2302427 variant located in the 185th amino acid of EZH2 with an amino acid change of Asp to His. The PolyPhen-2 (http://genetics.bwh.harvard.edu/pph2/) predicts that this variant have a 60.3% possible to influence EZH2 based on physical and comparative considerations. This variant was also predicted to have function with a AA change score being 85 in MutationTaster2 (score ranging from 0-215).

## DISCUSSION

This is the first study of genetic polymorphism in exon region of EZH2 and risk of colorectal cancer. Through a case control study consist of 852 cases and 1,303 controls of Chinese population, we identified that rs2302427 (Asp185His) was significantly associated with risk of colorectal cancer. We found that the frequency of GC and CC genotypes of rs2302427 were significantly lower in CRC patients than the healthy controls. Moreover, through a stratification analysis, the significant association was found only in younger patients and smokers, but not in older patients or nonsmokers. These results indicated an important role of EZH2 in CRC carcinogenesis.

EZH2 encodes a member of the Polycomb-group (PcG) family, function as a subunit of polycomb repressor complex 2 (PRC2), which involved in maintaining the transcriptional repressive state of genes over successive cell generations. PRC2 catalyses the mono-, di- and trimethylation of lysine 27 of histone H3 (H3K27me1, H3K27me2 and H3K27me3, respectively) [12]. H3K27me3 is a hallmark of transcriptional silencing and is thought to result in gene repression together with PRC1, which play important roles in cancer [21, 22]. EZH2 was initially shown to have oncogenic functions; however, many studies in recent years has shown that PRC2 also has tumor suppressive effects in cancer [23]. EZH2 has also been considered as a biomarker for multiple cancers [24]. Polymorphisms in EZH2 has also been reported to be associated with multiple cancer, including colorectal cancer [17-20]. In this study, we found a novel missense variant (rs2302427) that was correlated with decreased risk of colorectal cancer. A previous study has proved that rs2302427 variant decreased histone methyltransferase activity of EZH2, making this association biological possible [25, 26].

Our study also has some limitations. First, the study has constrained by a relatively small sample size that we did not investigate rare missense variants in EZH2. Second, the present case-control study only tested in one center. More replication studies should be performed to confirm the association between rs2302427 and risk of CRC in the future. Third, although previous studies have proved that rs2302427 may influence EZH2, more functional experiments should be performed to investigate how this variant affect CRC susceptibility. Third, the conclusion that the variant was only associated with risk of colorectal cancer in younger patients and smokers need to be confirmed in larger samples.

In summary, through screen single nucleotide polymorphisms (SNPs) in the exon region of EZH2 gene, we found that rs2302427 is associated with susceptibility to CRC. Compared to rs2302427C allele, individuals with rs2302427G was associated with lower CRC risk, especially in younger patients and smokers. This study provided new evidence for the precise medicine of CRC.

## MATERIALS AND METHODS

### Study subjects

CRC patients were enrolled from Shandong Cancer Hospital Affiliated to Shandong University, Jinan, China between January 1st, 2014 to August 30th, 2016. Controls samples were selected from a community cancer screening program for early detection conducted in the same region during the same period as cases were collected. All CRC patients were confirmed primary CRC, without any radiotherapy or chemotherapy treatment prior to blood samples collected. Both cases and controls were Han Chinese descent. The informed consent was obtained from every participant at recruitment and peripheral blood samples and demographic characteristics such as gender, age and ethnicity were collected by interviewers. This study was conducted under the approval of the institutional review boards of Shandong Cancer Hospital Affiliated to Shandong University.

### SNP selection and genotyping

We searched for variants in the exon region of EZH2 with minor allele frequency (MAF) > 0.01 in Chinese Han Beijing (CHB) population. One variant (rs2302427, TCATCATCGT [C/G] ATCATCATTA in forward strand) was selected and was genotyped in a Chinses population consist of 852 cases and 1,303 controls. Genomic DNA were extracted from at least 1 ml peripheral blood sample collected from each participant at recruitment. Genotyping were performed using Taqman SNP Genotyping Assay (Applied Biosystems). The case and control samples were mixed in the plates, and persons who performed the genotyping assay were not aware of case or control status.

### Statistical analysis

Unconditional multivariate logistic regression analysis with adjustment of sex, age, smoking status and drinking status was performed to assess the association between rs2302427 and risk of CRC. All statistical analyses were performed using R software. All tests were two-sided and *P* < 0.05 were considered significant.

## Abbreviations

Colorectal cancer: CRC
Enhancer of zeste 2 polycomb repressive complex 2 subunit: EZH2
Polycomb-group: PcG
Polycomb repressor complex 2: PRC2
Lysine 27 of histone H3: H3K27
Monomethylation of lysine 27 of histone H3: H3K27me1
Dimethylation of lysine 27 of histone H3: H3K27me2
Trimethylation of lysine 27 of histone H3: H3K27me3
Single nucleotide polymorphisms: SNPs
Chinese Han Beijing: CHB
